# Positionality statements in science

**DOI:** 10.12688/openreseurope.17058.2

**Published:** 2024-06-21

**Authors:** Veli-Matti Karhulahti

**Affiliations:** 1 University of Jyväskylä, Jyväskylä, Finland

**Keywords:** Bias. Epistemology, Interdisciplinary, Knowledge, Methodology, Objectivity, Philosophy of Science, Subjectivity

## Abstract

The goal of this essay is to clarify positionality as an epistemological scientific concept and address related misunderstandings to help researchers assess whether statements thereof contribute to their work. Positionality statements can be useful for various research designs across scientific fields, when they are used knowingly.

## Introduction

Different branches of science are sometimes compared to games that are played by slightly different rules ^1^. In interdisciplinary research and multidisciplinary journals, it can be difficult for editors, reviewers, and readers to assess whether scientists have correctly followed the rules because multiple games are played simultaneously. One of the rules that has recently confused the scientific world concerns *positionality statements,* which refer to disclosing the scientist’s position in relation to the conducted research. While positionality has historically and pragmatically established functions in certain methodological traditions ^2– 4^, in the past few years, an increasing number of scientists across disciplines have started disclosing their positions ^5– 7^. This essay aims to clarify positionality as an epistemological concept and research tool to help scientists in all fields decide whether such statements can contribute to their work.

## What positionality does

Some methodologies operate with data and analyses that are mostly visible, whereas others do not reach transparency to the same degree. The epistemological treatise of positionality originates mainly from the latter, such as qualitative research traditions, where both producing and analyzing data depend heavily on the tacit interactions of individual researchers ^3, 4^. A statement of the researcher’s position can serve as metadata that sheds light on both data generation and analysis.

Reflecting on the scientist’s position has long been a central part of anthropological and other interpretively oriented research paradigms ^2, 3^. Although positionality can be related to biases, it is not a matter of bias *per se* but a much larger question of calibrating the research process. When the scientist itself is part of the toolset that is used to generate primary research data—for instance, ethnographic fieldnotes—the details of that “tool” are relevant to acknowledge, not unlike recognizing and transparently reporting materials in biology, physics, or psychology. Because engaging in fieldwork is a process where only a small part of interactions and observations can be documented, it matters *who* engaged and selected the fieldnote data. In these cases, reflecting on the relevant aspects of the scientist is part of the research process and also makes the scientific community, including meta-analysts, better equipped to assess the study and its components.

Compared to quantitative analytic traditions, qualitative research methods often depend heavily on the scientist’s interpretive effort and are thus even more flexible when it comes to researcher degrees of freedom ^8^. Positionality statements can provide contextualizing information that explains some of the differences between two or more interpreters. For example, a non-Korean scientist who manually codes medical interview data from South Korea may be unable to identify signals that are evident to Korean scientists. Meanwhile, the same non-Korean scientist, as an “outsider,” may be positioned to see other meanings more clearly. Reflecting on such positions can serve as a helpful analytic tool, especially in proactive use. As in preregistration more generally ^9^, stating pre-study assumptions can help making useful analytic calibrations before it is too late. In registered reports, positionality statements may also help inform early peer feedback ^10^.

## Two common misunderstandings

A common misunderstanding is that scientific positionality statements must pick a set of group identities, as from a ‘shopping list’ ^11^. Nonetheless, there are no reasons to expect all group identities to be relevant for the generation and analysis of all research questions. Each positionality statement should fit the methods and topics of a specific study: What are the scientists’ backgrounds, beliefs, and other aspects that are likely to affect the generation or analysis of these *particular* data? In a psychiatric interview study, the scientist’s (lack of) clinical background is likely to be relevant, whereas an ethnographic study of gender interactions may benefit from reflecting on the scientist’s gender ^4^. Because it can never be perfectly known what aspects of a position meaningfully affect the data and analyses, pre-study reflections on positionality serve essentially as auxiliary hypotheses: explicit assumptions about how a scientist’s subjectivity can affect upcoming findings.

The second enduring misunderstanding is that positionality rests on standpoint epistemology or postmodernism, which may sometimes even challenge the possibility of scientific knowledge ^2, 6^. Reflections of positionality do not demand a specific epistemology, yet epistemological (and ontological) assumptions can belong to a stated position. As the earlier examples illustrate, positionality statements can contribute to values that align with objectivity and traditional philosophies of science, such as those based on falsification ^12^. Nonetheless, positionality statements should always be used *knowingly* with a function that serves the study in question. For example, multiauthor studies that operate with numerous experts may benefit more from closed-ended position surveys than from hundreds of statements. For research designs that deal with thematic political analyses by one or two authors, detailed statements of positionality can be advantageous. Because positionality does not equally benefit (or harm) all studies, journals should not mandate positionality statements but treat them like other research tools: encourage them to be used wisely when fitting with the methodology and philosophy of a study.

## To position or not

How should scientists decide when to use a positionality statement? The first step is to reflect on one’s own role within the chosen study and its topic: the more weight a research design has on individual scientists and their implicit decisions or interactions, the more likely a position is to impact the results, especially if the research question has significant subjective relevance. Although the role of individual scientists, for example, in controlled statistical designs may be minor, positionality statements can contribute to such research too. The increasing number of adversarial collaborations and unresolved disputes over meaningful effect sizes demonstrate how positions may also link to statistical decisions as well as inference ^13^. Positionality is not a bias, but can turn into one if a scientist fails to acknowledge its true effects.

Second, the relevance of positionality does not differ from that of setting other assumptions for a study. Reflexive self-assessments will sometimes produce false negatives and positives, as do other assumptions, but the very act of transparent reflection already contributes to useful critical positioning. Openly reflecting on one’s own role in the research process subjects it to the same scrutiny as other research materials ^14^. In the same way as it is impossible to guarantee that reported data exclusions, manipulations, and other research decisions correspond to reality ^15^, the degree to which positionality statements echo actual positions can never be fully known. Nonetheless, spelling out positionalities and their (lack of) relevance remains one of the tools that can help scientists across fields not fool themselves, as long as the tool is used knowingly.
