# Supplementary material for: Positionality statements in science
Source: Open Res Eur. 2024 Jun 21;4:62. Originally published 2024 Mar 27. [Version 2] doi: 10.12688/openreseurope.17058.2 (PMC11200055; doi:10.12688/openreseurope.17058.2)
Supplement: Peer review history

# Reviewer report: Reviewer response for version 2

**DOI:** 10.21956/openreseurope.19542.r41568

**Published:** 2024-06-25

**Authors:** Ingo Rohlfing

**Affiliations:** 1 University of Passau, Passau, Germany

I appreciate the author's feedback. The revisions are fine to me. The article is in good shape now.

Is the argument persuasive and supported by appropriate evidence?

Yes

Is the work clearly and cogently presented?

Yes

Is the topic of the essay discussed accurately in the context of the current literature?

Yes

Does the essay contribute to the cultural, historical, social understanding of the field?

Yes

Reviewer Expertise:

social science research methods; meta science; open science

I confirm that I have read this submission and believe that I have an appropriate level of expertise to confirm that it is of an acceptable scientific standard.

# Reviewer report: Reviewer response for version 1

**DOI:** 10.21956/openreseurope.18432.r40176

**Published:** 2024-05-30

**Authors:** Nicki Lisa Cole

**Affiliations:** 1 Know-Center GmbH, Graz, Austria

This essay discusses the concept of positionality, the practice of writing and publishing positionality statements, and what ends this practice serves. The essay is well situated in relevant literature, clearly and persuasively written and backed by evidence, and offers a valuable contribution to the methodological cannon for interpretive research traditions, and for scientific methodology more broadly. My only critique, is that it could be explicitly stated that reflecting on positionality is not only useful and important within interpretive/qualitative positions, but can also be for scientific research, generally speaking.

Is the argument persuasive and supported by appropriate evidence?

Yes

Is the work clearly and cogently presented?

Yes

Is the topic of the essay discussed accurately in the context of the current literature?

Yes

Does the essay contribute to the cultural, historical, social understanding of the field?

Yes

Reviewer Expertise:

Qualitative research methodologies, open and reproducible research

I confirm that I have read this submission and believe that I have an appropriate level of expertise to confirm that it is of an acceptable scientific standard.

# Response

**Published:** 2024-06-16

**Authors:** Veli-Matti Karhulahti

Thank you for making time to review the essay. I appreciate the kind feedback and agree it would be valuable to explicitly state that positionality statements can be useful across sciences in general. For the second version, I have added this information both to the abstract and at the end of the essay.

# Reviewer report: Reviewer response for version 1

**DOI:** 10.21956/openreseurope.18432.r40170

**Published:** 2024-05-13

**Authors:** Ingo Rohlfing

**Affiliations:** 1 University of Passau, Passau, Germany

The submission is situated in work on positionality statements and the role of assumptions for empirical research. I see the main contribution as three-fold. The first and second contribution is the clarification of two misunderstandings. One is the belief that positionality is tied to set of group identities. The second misunderstanding relates to the idea that positionality statements have to be rooted in interpretivist or radical postmodern epistemologies and philosophies of science. The third contribution, which is related to the other two, is the argument that positionality statements involve assumptions about how the own experiences, knowledge etc. influence the empirical analysis.

 I find the submission valuable because it argues convincingly that the scope of positionality statements is larger than some or many researchers may think. I like the argument that preregistered research and many-analysts studies, which are primarily quantitative, would also benefit from positionality statements. They may help in explaining why researchers who try to answer the same research question with the same data arrive at different, sometimes contradictory results.

 The only point I am not convinced of is the analogy to games with imperfect information. I understand why information about an empirical analysis may be imperfect when a positionality statement is missing. However, I am not sure what the game is that is played. Who are the players? What are their utility functions? What are the players’ strategies? As I read it, the reference to games is not central to the paper. I suggest it is dropped, or elaborated on in more detail.

Is the argument persuasive and supported by appropriate evidence?

Yes

Is the work clearly and cogently presented?

Yes

Is the topic of the essay discussed accurately in the context of the current literature?

Yes

Does the essay contribute to the cultural, historical, social understanding of the field?

Yes

Reviewer Expertise:

social science research methods; meta science; open science

I confirm that I have read this submission and believe that I have an appropriate level of expertise to confirm that it is of an acceptable scientific standard, however I have significant reservations, as outlined above.

# Response

**Published:** 2024-06-16

**Authors:** Veli-Matti Karhulahti

Thank you for making time to review the essay. I appreciate the kind feedback and agree that the reference to imperfect information games was confusing and unnecessary. For the second version, I have simply omitted this part and added small clarifications about the qualitative-quantitative relationship in other parts of the essay.
